# Biphenotypic Sinonasal Sarcoma with Orbital and Skull Base Involvement Report of 3 Cases and Systematic Review of the Literature

**DOI:** 10.1007/s12070-023-03900-4

**Published:** 2023-06-22

**Authors:** Sofia Anastasiadou, Peter Karkos, Jannis Constantinidis

**Affiliations:** 1 Department of Ear Nose and Throat Surgery, ACHEPA Hospital, Thessaloniki, Greece; 2 Aristotle University of Thessaloniki, Thessaloniki, Greece

**Keywords:** Biphenotypic sinonasal sarcoma, Sinonasal tumours, Sinonasal malignancy, Sinonasal mass

## Abstract

Biphenotypic sinonasal sarcoma (BSNS) is a rare malignant tumour of the upper nasal cavity and ethmoid sinuses that presents predominantly in middle aged female patients and show a characteristic infiltrative and hypercellular proliferation of spindle cells that demonstrate a specific immunoreactivity. We present three cases with BSNS that had different presenting complaints, either sinonasal or orbital problems, underwent endoscopic surgical treatment and/or radiotherapy and have been disease free on long follow up. A systematic review of all published cases was performed to identify all BSNS cases known at present. BSNS requires prompt and correct diagnosis with accurate surgical resection as well as consideration of radiotherapy. Our three cases confirm the findings of the literature and support that BSNS is an aggressive but treatable malignant disease of the sinonasal tract.

## Introduction

There is a wide variety of sinonasal tumours with multiple presentations, phenotypes and symptoms. Sinonasal malignancies are a diagnostic and therapeutic challenge due to histologic diversity and proximity to vital structures like the orbit, skull base, brain and cranial nerves. Biphenotypic sinonasal sarcoma (BSNS) is one of the rarest and slow growing soft tissue sarcomas that has been only described in the last decade [1]. The morphologic features, the high recurrence rate but only locally without distant metastases and immunohistochemical and molecular findings demonstrate a highly differentiated tumour that requires thorough care [2]. The uniqueness of this tumour as well as its novelty requires further investigation and report of cases to ensure correct diagnosis and management in the future.

## Material and Methods

A thorough systematic review of the literature was conducted by two electronic databases Medline/Pubmed (1946–December 2022) and Embase databases (1947–December 2022) using the Ovid research tool. The research terms used were “biphenotypic”and “sinonasal” and “sinus” and “nasal” and “sarcoma” creating the MeSH terms respectively. A systematic review flowchart was created and followed to ensure coherence. Only 52 results were identified with the research terms described and abstract assessment led to inclusion of 27 articles that were either case reports or reviews of the existing literature. Studies or case reports that had doubtful results or did not confirm BSNS diagnosis were excluded from the search. Non English language articles were excluded from the search. Table 1 summarises the BSNS cases identified in the literature with main findings and key points of their age/gender, presentation and symptoms, site of lesion and extension, treatment modalities, recurrence rates and main genetic findings.

**Table 1:** Systematic review of the literature of BSNS

	References	Type of article	No of patients	Age	Gender	Symptoms	Site	Treatment	Follow up	Genetic Analysis
1	Bartoš et al. [3]	Case report	1	78	Female	Nasal congestion	Left middle meatus/ ethmoid cells	Endonasal resection	No information given	PAX3::MAML3 fusion
2	Nichols et al. [4]	Case report	1	54	Male	Nasal mass—headaches	Right middle turbinate/ethmoid/sphenoid sinus	Endoscopic surgery	No recurrence on 6 weeks post-op	PAX3::FOXO6 fusion protein
3	Hasnie et al. [4]	Case report	1	72	Female	Nasal congestion	Bilateral lamina papyracea/skull base/frontal sinuses	Endoscopic and bicoronal approach	Infection of the pericranial flap, pneumocephalus, and death	*PAX3-*MAML3 fusion (more aggressive)
4	Sudabatmaz et al. [5]	Case report	1	55	Female	Nasal congestion and facial pressure	Right middle meatus/ethmoid/sphenoid sinus	Endoscopic surgery	No recurrence on 1 year follow up	PAX3 gene not evaluated
5	Baneckova et al. [6]	Report of 3 cases and review of the literature	1 case with BSNS	41	Female	Not mentioned	Nasal cavity	Not mentioned	No recurrence on 8 years follow up	PAX3-MAML3 fusion not observed
6	Georgantzoglou et al. [7]	Case report	1	62	Female	Nasal congestion, epistaxis and shortness of breath	Left nasal cavity extending to ethmoid/sphenoid/maxillary sinuses/cribriform plate/ periorbital fat	Not mentioned	Not mentioned	PAX3::MAML3 but also PAX7, PAX8
7	Bell et al. [8]	Case report	1	66	Male	Left eye swelling, diplopia and nasal discharge	Intracranial and intraorbital extension	Bifrontal craniotomy and transnasal approach + chemoradiotherapy	Recurrence 15 years later	PAX3 gene not evaluated
8	Sethi et al. [9]	Report of 3 cases and review of the literature	3	55 43 70	Female Female Female	Left nasal congestion and headaches Right nasal obstruction Incidental finding	Left nasal cavity/ethmoid/sphenoid/ maxillary sinuses, cribriform plate/periorbital fat Right nasal cavity/ethmoid/sphenoid/maxillary sinuses/periorbital fat Not mentioned	Endoscopic surgery and radiotherapy Endoscopic surgery Endoscopic approach	No recurrence 32 months post-op Lost on follow up No recurrence 13 months post-op	PAX3 gene not evaluated *PAX3-*MAML3 fusion Not mentioned
9	Kominsky et al. [10]	Case report and review of the literature (100 cases)	2	60 70	Male Male	Nasal congestion and blurred vision bilaterally Nasal congestion and facial pressure	Left ethmoid sinus/ bilateral paranasal sinuses/left lamina papyracea Left frontal sinus/ethmoid cells/cribriform plate	Endoscopic surgery with duroplasty Endoscopic surgery with a second intervention to clear up margins	No recurrence 1 year post-op No recurrence 14 months post-op	Not mentioned Not mentioned
10	Hanbazazh et al. [10]	Case report	1	50	Male	Left diplopia, mild proptosis and nasal congestion	Left ethmoid sinus/lamina papyracea/periorbital fat	Left endoscopic removal of the intranasal tumour and Lynch-type orbital approach	Recurrence in 6 months follow up followed by craniotomy and radiotherapy–no recurrence	PAX3 rearrangement
11	Gross et al. [11]	Review of the literature	Not mentioned	Not mentioned	Not mentioned	Not mentioned	Not mentioned	Not mentioned	Not mentioned	Not mentioned
12	Miglani et al. [12]	Case series and Review of the literature	5	Median age 56 years	4/5 patients: Female	Not mentioned	5/5: Nasal cavity, 1/5: skull base 2/5: lamina papyreacea 1/5: medial rectus muscle	3/5: Open bifrontal craniotomy 2/5: Endoscopic resection Radiotherapy offered but refused	2/5: Unifocal recurrence–repeat surgical excision and radiotherapy	PAX3 rearrangement
13	Le Loarer et al. [13]	Case series	41	Median age 49 years	61% Female	Nasal congestion	68%: Nasal cavity 49%: ethmoid sinuses 27%: both 17%: facial sinuses 15%: erosion of bones	78%: Surgery 21%: Radiotherapy 4%: chemotherapy 4%: chemoradiotherapy	32%: Local recurrence at 9–95 months follow up	90%: PAX3-MAML3 fusion, 1 case PAX3-FOXO1 fusion 1 case: PAX3-WWTR1 fusion
14	Dean et al. [14]	Review of Imaging	Not mentioned	Not mentioned	Not mentioned	Not mentioned	Not mentioned	Not mentioned	Not mentioned	Not mentioned
15	Chitguppi et al. [1]	Case report and literature review	1 Literature review 95 cases	53 Mean age: 52 years	Male Female to male ratio 2.2/1	Nasal congestion and anosmia	Nasal cavity/Frontal and ethmoid sinuses/ orbit/skull base 28%: extrasinonasal extension	Endoscopic surgery and second stage transconjuctival surgery for intra-orbital part and Radiotherapy 1 case: Open surgery 1 case: endoscopic surgery With or without radiotherapy	No recurrence 32% recurrence in 1–28 years follow up	PAX3-MAML3 fusion PAX3-MAML3 fusion
16	Sugita et al. [15]	Case report	1	30	Female	Left nasal congestion and orbital swelling	Ethmoid sinus	Combined endoscopic and transcranial approach and radiotherapy	No recurreence on 3 months follow up	PAX3-MAML3 fusion
17	Alkhudher et al. [16]	Case report	1	35	Female	Right nasal obstruction and epistaxis	Nasal cavity/ septum/ medial maxillary wall	Endoscopic surgery	No recurrence on 2 years follow up	Not mentioned
18	Carter et al. [2]	Literature review	No totals provided due to overlap of cases	Mean age 50–51 years	Female to Male ratio: 2/1	Nasal obstruction/epistaxis/sinus pain	Sinonasal expansion/orbit(25%), skull base (10%)	Surgical removal with or without radiotherapy	40–50% Recurrence rate (1–9 years follow up), 1 reported death	Not mentioned
19	Andreasen et al. [17]	Literature review	55 cases (41 genetically characterised)	Median age: 47 years	Female to Male ratio: 2/1	Nasal congestion	Ethmoid/frontal/maxillary/sphenoid sinus involvement	Surgical removal either endoscopic or open	2%: 3 local recurrences, 31% local recurrence in 64–72 months follow up	7%: PAX3-MAML3 fusion, 19%: PAX3 rearrangement
20	Fudaba et al. [18]	Case report	1	70	Male	Haematemesis and low GCS	Left frontal sjull base and ethmoid sinus	Combined transcranial and endoscopic surgery	Recurrence after 11 years (post endoscopic removal)	No PAX3 rearrangement
21	Kakkar et al. [19]	Case series	6	Mean age 51 years	Male to Female ratio 1/5	Nasal obstruction	83% nasal cavity 17% ethmoid sinus and base of skull	50% lateral rhinotomy and excision 50% refused treatment or lost follow up	34% recurrence locally, 17% no recurrence on 10–18 months follow up, no recurrence on recently operated patient	No PAX3 assessment, but β-catenin assessment (not specific for BSNS)
22	Zhao et al. [20]	Case series	4	Median age 35 years	Male to Female ratio 2/1	Nasal congestion	Not mentioned	Not mentioned	No recurrence on 3–15 months follow up	PAX3-FOXO1 rearrangement
23	Lin et al. [21]	Case report	1	67	Female	Right nasal obstruction and mass	Right nasal cavity/maxillary/fontal sphenoid/ethmoid sinuses/ skull base and frontal brain lobe	Endoscopic surgery with craniofacial resection	Death perioperatively	Peak apart signal of PAX3
24	Fritchie et al. [22]	Case series	44 BSNS samples (4 patients assessed)	Median age 40 years	Male to Female ratio 1/1	Not mentioned	75% nasal cavities, 25% skull base	Not mentioned	50%:No recurrence on 12 months follow up 25%: local recurrence unclear 25%lost on follow up	55% PAX3-MAML3 fusion, 34% PAX3 rearrangement 6% PAX3-FOXO1 fusion, 9% no PAX3 involvement
25	Rooper et al. [23]	Case series	11	Median age 44 years	Male to Female ratio 3/8	Not mentioned	100%: Upper sinonasal tract 25%: ethmoid sinus 20% frontal sinus 20% all sinuses 15% orbital extension	Not mentioned	71% No recurrence on 12–26 years follow up 28% local recurrence	PAX3 not assessed properly due to possible technical failure
26	Huang et al. [24]	Case series	7	Median age 47	Male to Female ratio 4/3	Not mentioned	40% Frontal/ ethmoid sinuses 20% Nasal cavity 20% both	87% Surgery alone 13% surgery with chemoradiotherapy	Follow up in 4 cases: 1 case: local recurrence on 3 year follow up	50% PAX3-NCOA1 fusion 50%PAX3-MAML3 fusion
27	Wong et al. [26]	Case report	1	33 years	Male	Epistaxis	Left nasal cavity/ left sphenoid sinus	Endoscopic removal with chemoradiotherapy	No recurrence on 5 months follow up	PAX3-FOXO1 fusion

*PAX3* Paired box family–3 gene; *MAML3* Mastermind like transcription coactivator activity; *FOXO1* Forkhead box transcription factor; *WWTR1* WW domain containing transcription regulator 1

## Cases

### Case 1

**Fig. 1:**
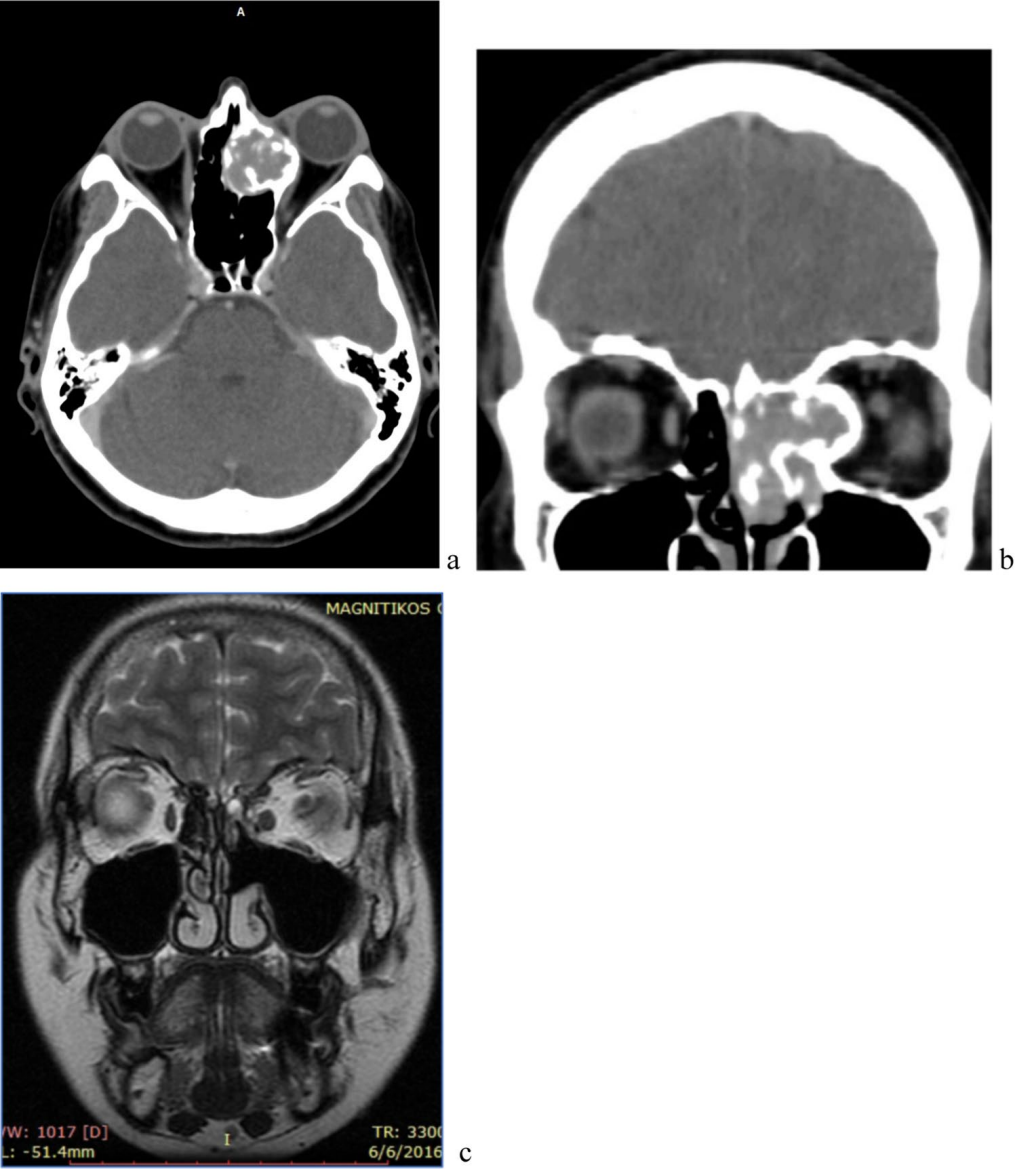
Pre-operative CT scan, axial slice (**a**) and coronal slice (**b**) (Case 1), **c** Post-operative MRI scan (Case 1)

We present a 52 year old lady that suffered with exophthalmos symptoms and was initially assessed by ophthalmology specialists. During her investigation process, she underwent a CT scan of her orbits and sinuses that revealed an ossified hard tumour extending from the sinonasal tract until the cribriform plate and into the orbit, pressing the rectus medialis and the orbital fat causing exophthalmos (Fig. 1a, b). Office biopsy was most consistent with a low-grade spindle cell carcinoma. An endoscopic resection of the tumour was performed adopting the cavitation technique for complete removal of the mass. More specifically, after tumor debulging a middle meatal antrostomy, and complete ethmoidectomy provided access into the intraorbital part. The middle turbinate was removed to ensure free margins of the histological specimen. The ossification of the tumour worked as an adjunct towards its complete removal and there was also reassurance of the clear margins of the resection in the histology report that prevented this lady from having post-operative radiotherapy. Final pathology returned as BSNS characterized by a low cellular proliferation of spindle cells arranged in interwoven fascicles with major calcification. She was therefore followed up with a MRI scan of her orbits and sinuses that showed clearance of the disease observed 7-year postoperatively (Fig. 1c).

### Case 2

**Fig. 2:**
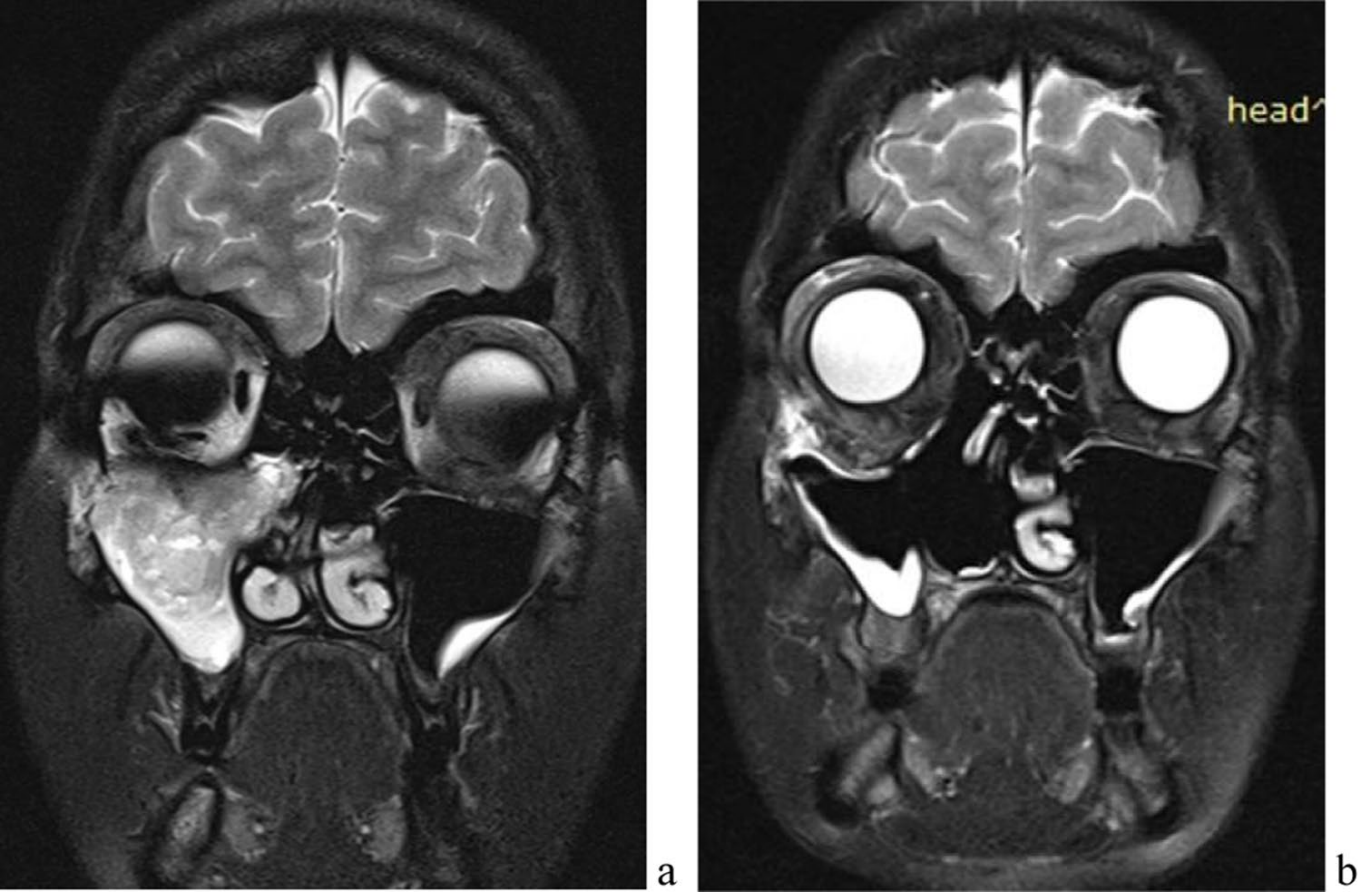
Pre- and postoperative MRI scan (Case 2)

Α 30-year old lady who presented with pressure symptoms of her orbit resulting in bulb protrusion without any other complaints. She underwent a CT scan of her orbits and sinuses that revealed a tumour in the maxillary sinus with orbital floor destruction and intraorbital extension (Fig. 2a). Primary biopsy was most consistent with a low-grade spindle cell carcinoma. An endoscopic resection of the tumour was performed via a medial maxillectomy and anterior ethmoidectomy. Intra-operative frozen sections from adjacent anatomical structures were negative for malignancy. Final pathology revealed a BSNS characterised by moderate to highly cellular proliferation of spindle cells arranged with focal rhabdomyoblastic differentiation. This lady had subsequent radiotherapy since at the time of the diagnosis the tumour had already spread into the orbit and the resection was challenging due to the proximity to the infraorbital nerve that was eventually preserved. She was also followed up with MRI scan that 6 years after her surgery show no local recurrence of the disease (Fig. 2b).

### Case 3

**Fig. 3:**
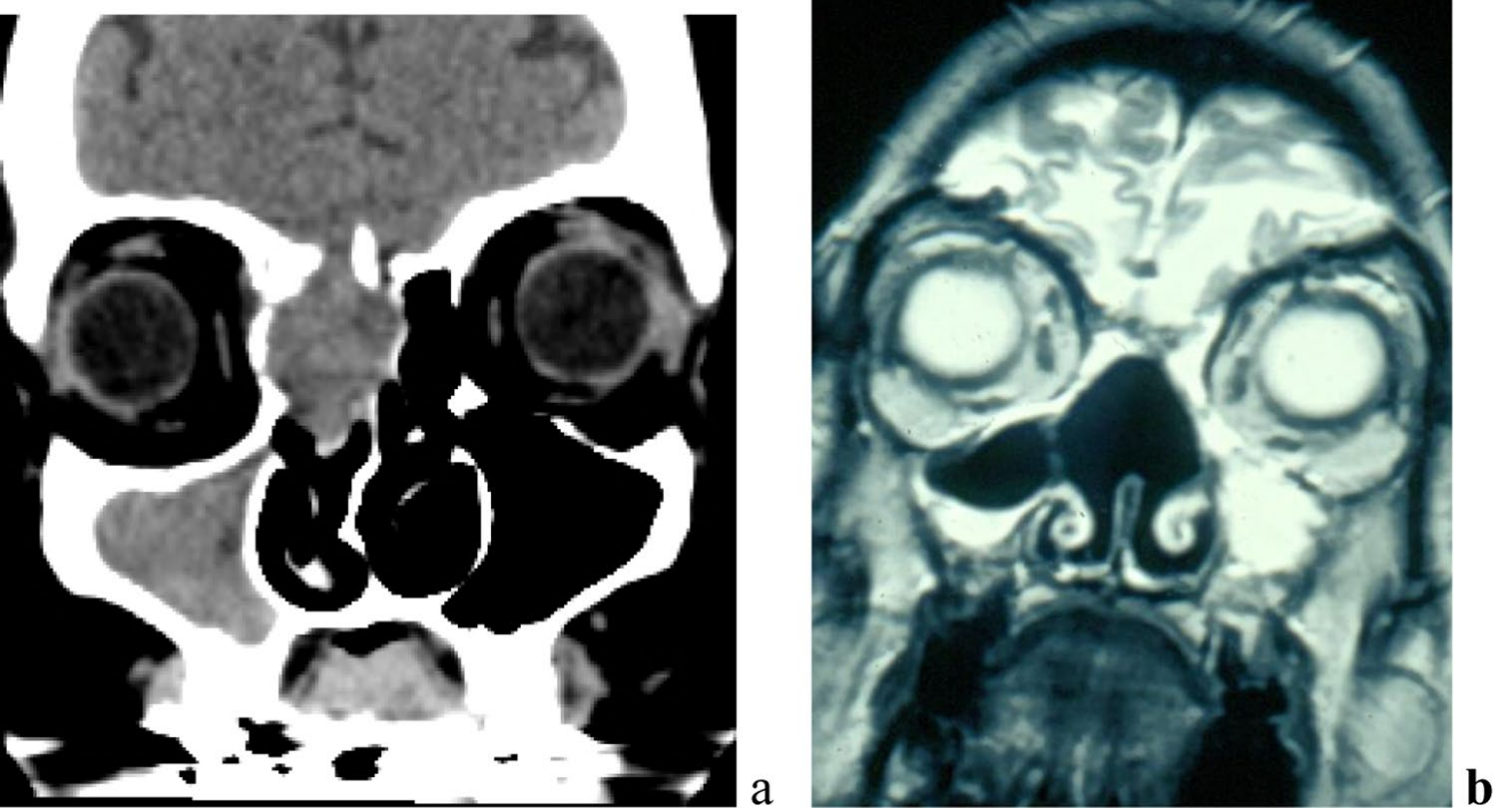
Pre-operative CT scan (**a**) and postoperative MRI scan (**b**) (Case 3)

The third case is summarised in a 47 year old lady who presented with severe recurrent headaches as her main concern. She underwent a CT scan of her paranasal sinuses that revealed a mass extending in the middle meatus involving the orbit and the anterior skull base (Fig. 3a). Primary biopsy was most consistent with a low-grade spindle cell malignant tumor. An endoscopic transnasal approach was used for gross tumor resection. Intraoperatively, the mass was found to extend superiorly to involve the cribriform plate, medially the nasal septum and was laterally adherent to the lamina papyracea. After unilateral middle meatal antrostomy, complete ethmoidectomy on both sites, the superior nasal septum was removed and a type three drainage of the frontal sinus was performed. The lamina papyracea of tumor site was removed and the cribriform plate and crista galli were resected, resulting in a small dural defect with a low flow cerebrospinal fluid (CSF) leak. This was repaired with a fascia lata graft and a local mucoperiostal flap from the contralateral septum which can be rotated to resurface the skull base defect (flip-flap). Intraoperative margins returned negative. Histopathology of the tumor confirmed diagnosis of BSNS. The patient was also decided to have radiotherapy to complete her treatment since the disease was progressed at the time of diagnosis. In her 4-year follow up, she appears to be disease free and has no headaches or other sinonasal symptoms (Fig. 3b).

## Discussion

Sinonasal tract tumours are neoplasms that affect mostly the sinuses, internal nasal cavities, orbits, skull base and in some cases can have intracranial extension. Common presenting symptoms are nasal obstruction, epistaxis, facial pressure or pain, smell impairment, as well as neurological or ophthalmic complaints due to the tumour’s extension [25, 26]. In our cases, the main symptoms were headaches or macroscopic changes of the eye orientation. The diversity of sinonasal tumours makes their identification and diagnosis challenging due to the large spectrum of their clinical presentation as well as the the histopathological origin that can be neurogenic, myogenic, fibroblastic, vascular or can even reveal benign reactive proliferation.

Biphenotypic sinonasal sarcomas were firstly discovered by Lewis et al. in 2012 [27], who described them as low grade spindle sarcomas of the sinonasal tract. WHO announced addition of this entity in the reviewed 2017 WHO classification of head and neck tumours including BSNS as one of the newly discovered tumours of the sinonasal cavity [28–30]. These tumours have double neural and myogenic differentiation but are histologically different from malignant sarcomas or other sinonasal cancerous masses. The primary different characteristic of this group is the biphenotypic marker expression during the immunohistochemical analysis as well as its unique identity combining clinical, morphologic, histologic and genetic features. In our cases, all three patients presented with generic sinonasal symptoms and initially underwent routine investigations, primarily CT and MRI scan of their orbits and sinuses as well as flexible nasoendoscope to assess the nature of the sinonasal masses. Although the above are all adjuncts to a thorough surgical planning for mass excision, they have minimal to offer towards determining the diagnosis. In all BSNS cases, imaging modalities and endoscopic investigations reveal an enhancing soft tissue mass with infiltrative growth associated with hyperplastic bone or even bone infiltration. It is therefore evident that minimal features exist to guide the ENT surgeon towards BSNS as these entities present similar to other nerve sheath tumours, mesenchymal neoplasms and other varieties of sarcomas [31]. It is therefore histological, immunochemical and genetic analysis which is required to confirm diagnosis of BSNS.

BSNS histopathological analysis reveals a spindle cell carcinoma that infiltrates the surrounding tissues including the nasal bones [32]. It is mostly unencapsulated and macroscopically gives the impression of a polypoid mass [13]. Microscopically, the spindle cells are organised in fascicles with all nuclei arranged in the same direction mimicking a herringbone pattern. There are no foci and tumour cells mostly lie on a fragile collagenous matrix with minimal mitotic activity[9]. In our cases, all three tumours had macroscopic features of a normal polyp with no ulceration, haemorrhage or necrosis and no characteristics of malignancy while erosion of bone and destruction of surrounding tissues was observed on imaging modalities. The histopathological analysis showed BSNS with small sinonasal-type glands, lymphocytes and macrophages with an increased vascular network, features that are typical of BSNS according to the literature.

However, the important finding that determined the diagnosis was smooth muscle actin (SMA) and S100 positivity with EMA and CD34 negativity during the immunohistochemical analysis [13, 24]. Normally, BSNS tumours show immunoreactivity for S100, SMA and sometimes desmin but demonstrate no reaction with CD34, STAT6, EMA and myogenin, which was also included in the report of all three cases presented. According to the literature and histological reports for BSNS mentioned in all 52 articles reviewed, it is evident that the BSNS is consistently positive for S100, calponin, actin, factor XIIIa and β-catenin; in some cases positive for myogenin, desmin, cytokeratins and EMA; and it is always negative for SOX10 [23, 33].

In terms of genetic analysis, PAX3 and MAML3 are genes involved in BSNS and their mutations may lead to different presentations of the disease or even different sinonasal mesenchymal tumours [17]. MAML3 is one of the mastermind-like (MAML) family of transcriptional co-activators that contribute to significant stages of cell life cycle such as cell proliferation, differentiation and death. Genetic analysis is performed using the FISH technique followed by PCR focusing on PAX3 re-arrangement atypias. PAX3 and MAML3 fusion is most commonly seen in BSNS while combinations such as *AX3-FOXO1, PAX3-MAML1, PAX3-MAML2, PAX3-NCOA1, PAX3-NCOA2 and PAX7-MAML3 are also observed. To make differential diagnosis more challenging, most of the above combinations exist in various sinonasal sarcomas such as the PAX3-FOXO1 and PAX3-NCOA1 that exist in rhabdomyosarcomas. However, the pathognomonic finding of PAX3-MAML3 fusion transcript is an adjunct towards BSNS diagnosis* [17, 34]. *Interestingly, it emerges from the literature that different combinations lead to various presentations of BSNS with characteristic tumour site or extension or even affecting the recurrent rates. In order to achieve such results though, more cases with genetic testing are required to gain safe and reliable information on how genetics affect clinical variations * [35]. Unfortunately, our histopathology team did not proceed to genetic testing, however BSNS has unique histological and immunohistochemical findings that lead towards the correct diagnosis as seen in various other cases in the literature where FISH genetic testing could not be performed.

Regarding treatment modalities, all cases in the literature were treated with surgical excision either endoscopic or open using craniotomy or lateral rhinotomy as an access point with or without adjuvant radiotherapy with some cases receiving chemotherapy as well (Table 1). Recurrence was observed in both groups irrespective of having adjuvant radiotherapy post operatively. There is therefore, no important evidence in the current literature to argue towards concomitant radiotherapy or surgical excision alone [5, 36]. In the literature, BSNS show significant extrasinonasal extension (approximately 27%) with the most common site of extension to be the cribriform plate. Local recurrence rate is considered high but fortunately, no distant metastasis was observed in any case with BSNS in the literature. In our cases, orbital and skull base involvement was observed, however radiotherapy was selected only according to intra-operative findings regarding the tumour infiltration of surrounding tissues. Despite having only endoscopic resection of the tumour that was invading the orbital fat, the patient in case 1 has no recurrence on their 7-year follow up, even without receiving adjuvant radiotherapy. It is therefore mindful to advocate, that radiotherapy should be individually selected in patients with spreading tumours and difficulties in complete endoscopic resections and should always be a result of multidisciplinary team discussion and involvement of patient views in the decision. The rarity of the disease and the small number of cases described in the literature limit the accurate assessment of treatment efficacy and more data is needed.

## Conclusion

Biphenotypic sinonasal sarcomas are uncommon low-grade spindle cell carcinomas in the sinonasal tract that demonstrate positive myogenic and neural differentiation. The clinical importance of these tumours is summarised to their common symptoms in association with the non-specific radiological findings but their high local recurrence rates that makes the early diagnosis and full treatment critical. Additional radiotherapy should always be a consideration but individualised treatment according to clinicopathological features of the tumour is key. Treatment with radiotherapy is individualised and is supported by concrete criteria based on location of the tumour, intraoperative surgical margins, histopathological features and general condition of the patient. It is therefore crucial for the multidisciplinary team that consists of the ENT surgeon, radiologist and primarily pathologist as well as oncologist, to be aware of this sinonasal entity to correctly diagnose BSNS, avoid misdiagnosis and treat effectively and successfully the disease.
